# Effects of Puerarin on the Ovariectomy-Induced Depressive-Like Behavior in ICR Mice and Its Possible Mechanism of Action

**DOI:** 10.3390/molecules24244569

**Published:** 2019-12-13

**Authors:** Ariyawan Tantipongpiradet, Orawan Monthakantirat, Onchuma Vipatpakpaiboon, Charinya Khampukdee, Kaoru Umehara, Hiroshi Noguchi, Hironori Fujiwara, Kinzo Matsumoto, Nazim Sekeroglu, Anake Kijjoa, Yaowared Chulikhit

**Affiliations:** 1 Division of Pharmaceutical Chemistry, Faculty of Pharmaceutical Sciences, Khon Kaen University, Khon Kaen 40002, Thailand; ariyawanps@gmail.com (A.T.); oramon@kku.ac.th (O.M.); onchuma.aim@gmail.com (O.V.); charkh@kku.ac.th (C.K.); 2 Department of Pharmacognosy, School of Pharmaceutical Sciences, University of Shizuoka, Yada 52-1, Shizuoka-shi, Shizuoka 422-8526, Japan; umehara@u-shizuoka-ken.ac.jp (K.U.); noguchi@u-shizuoka-ken.ac.jp (H.N.); 3 Division of Medicinal Pharmacology, Institute of Natural Medicine, University of Toyama, 2630 Sugitani, Toyama 930-0194, Japan; arawijuf@inm.u-toyama.ac.jp (H.F.); mkinzo@inm.u-toyama.ac.jp (K.M.); 4 Department of Horticulture, Faculty of Agriculture, Killis 7 Aralik University, Killis 79000, Turkey; nsekeroglu@gmail.com; 5 *ICBAS*-Instituto de Ciências Biomédicas Abel Salazar and CIIMAR, Rua de Jorge Viterbo Ferreira, 228, 4050-313 Porto, Portugal

**Keywords:** puerarin, *Pueraria candollei* var. *mirifica*, ovariectomy, forced swimming test, tail suspension test, neurogenesis

## Abstract

Daily treatment of ovariectomized (OVX) ICR mice with puerarin, a glycosyl isoflavone isolated from the root bark of *Pueraria candollei* var. *mirifica*, and 17β-estradiol attenuated ovariectomy-induced depression-like behavior, as indicated by a decrease in immobility times in the tail suspension test (TST) and the forced swimming test (FST), an increase in the uterine weight and volume, a decrease in serum corticosterone levels, and dose-dependently normalized the downregulated transcription of the brain-derived neurotrophic factor (BDNF) and estrogen receptor (Erβ and Erα) mRNAs. Like 17β-estradiol, puerarin also inhibited ovariectomy-induced suppression of neurogenesis in the dentate gyrus of the hippocampus (increased the number of doublecortin (DCX)-immunosuppressive cells). These results suggest that puerarin exerts antidepressant-like effects in OVX animals, possibly by attenuating the OVX-induced hyperactivation of the HPA axis and/or normalizing the downregulated transcription of BDNF and ER mRNA in the brain.

## 1. Introduction

Physiological fluctuations of estrogen in women are an important factor that affects mood, behavior, and cognition during their lifespan. A sharp decline in estrogen levels, which occurs during the anestrus cycle and menopausal transition, post-menopause, and surgical menopause, causes the most significant alterations in mood and cognitive function [1,2]. Thus, females exposed to a state of estrogen deprivation are prone to a higher risk of neurological symptoms, such as anxiety, depression, and memory impairment, than males.

Preclinical studies demonstrated that bilateral ovariectomy and aging make female rodents more susceptible to stressful stimuli than control animals, inducing emotional deficits such as anxiety and depression. These symptoms can be reversed by supplementation with estrogen. A body of evidence suggests that such estrogen replacement therapy (ERT) can exert anxiolytic and antidepressant effects in menopausal females by modulating the hypothalamic–pituitary–adrenal (HPA) axis functions [3,4]. However, the drawback of ERT is its adverse effects, such as an increased risk of breast and endometrial cancers, as well as thromboembolic events [5,6]. Consequently, the long-term usage of ERT to ameliorate emotional and cognitive deficits has an obvious limitation in women. Thus, the search for new phytoestrogens, with a weak effect on reproductive organs, from natural sources continues to be an important field of research.

**Figure 1:**
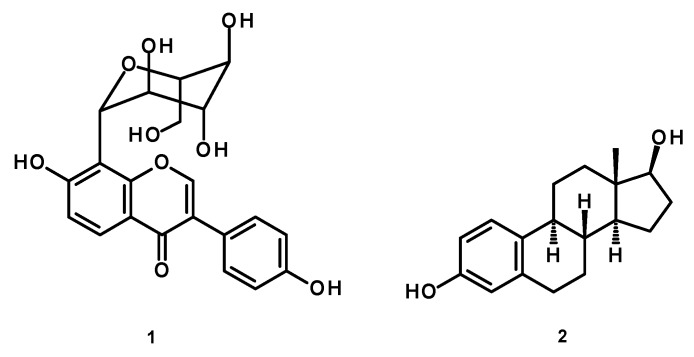
The structures of puerarin (**1**) and 17β-estradiol (**2**).

*Pueraria candollei* var. *mirifica* is a medicinal plant used in Thai traditional medicine for its rejuvenating and estrogenic effects [7]. For this reason, this plant has been widely investigated for its constituents and pharmacological activities. Peerakam et al. [8] found that the 95% ethanol extract of this plant contained puerarin (**1**), as a major compound, and other isoflavonoids, such as daidzin, daidzein, genistin, and genistein, in addition to the phytoestrogens miroestrol and deoxymiroestrol. Suthon et al. [9] evaluated the anti-osteoporotic effects of the crude extract of *P. candollei* var. *mirifica* and puerarin (**1**) in ovariectomized (OVX) mice and found that only the crude extract at 50 mg/kg (body weight) per day caused a significant increase in the trabecular bone mineral densities of tibia metaphysis when compared to the control group. On the other hand, the pharmacological activities of puerarin (**1**) (Figure 1), a major isoflavonoid from *P. candollei* var. *mirifica*, have been extensively investigated. Puerarin (**1**) has been widely used in the treatment of diabetes, dyslipidemia, liver fibrosis, cardiovascular diseases, neurotoxicity, and Alzheimer’s disease [10]. This compound was also found to exhibit various protective effects against fever, hyperlipidemia, osteonecrosis, inflammation, and oxidative damage [10,11]. Puerarin (**1**) was also investigated for its hypotensive effects and mechanisms in spontaneously hypertensive rats [12]. The effect of puerarin (**1**), isolated from *P. lobata*, on angiotensin II-induced aortic aneurysm formation was also investigated [13]. Although puerarin (**1**) was reported for its antidepressant-like effects in the chronic unpredictable stress-induced rats [14], its effects on depressive-like symptoms and neurogenesis in OVX mice, the animal model for menopause, have never been investigated. Therefore, the present study aimed to investigate the effects of puerarin (**1**) on depressive-like behavior in OVX mice, as well as to elucidate the underlying mechanisms related to HPA axis function, using 17β-estradiol (**2**) as a reference hormone. Additionally, the expressions of mRNAs of estrogen receptor (ERα and ERβ), brain-derived neurotrophic factor (BDNF), and doublecortin (DCX), which is an indicator/marker of neurogenesis in the hippocampus, were also examined.

## 2. Results

### 2.1. Effects of Puerarin (***1***) and 17β-Estradiol (***2***) on Ovariectomy-Induced Depressive-Like Behavior

**Figure 2:**
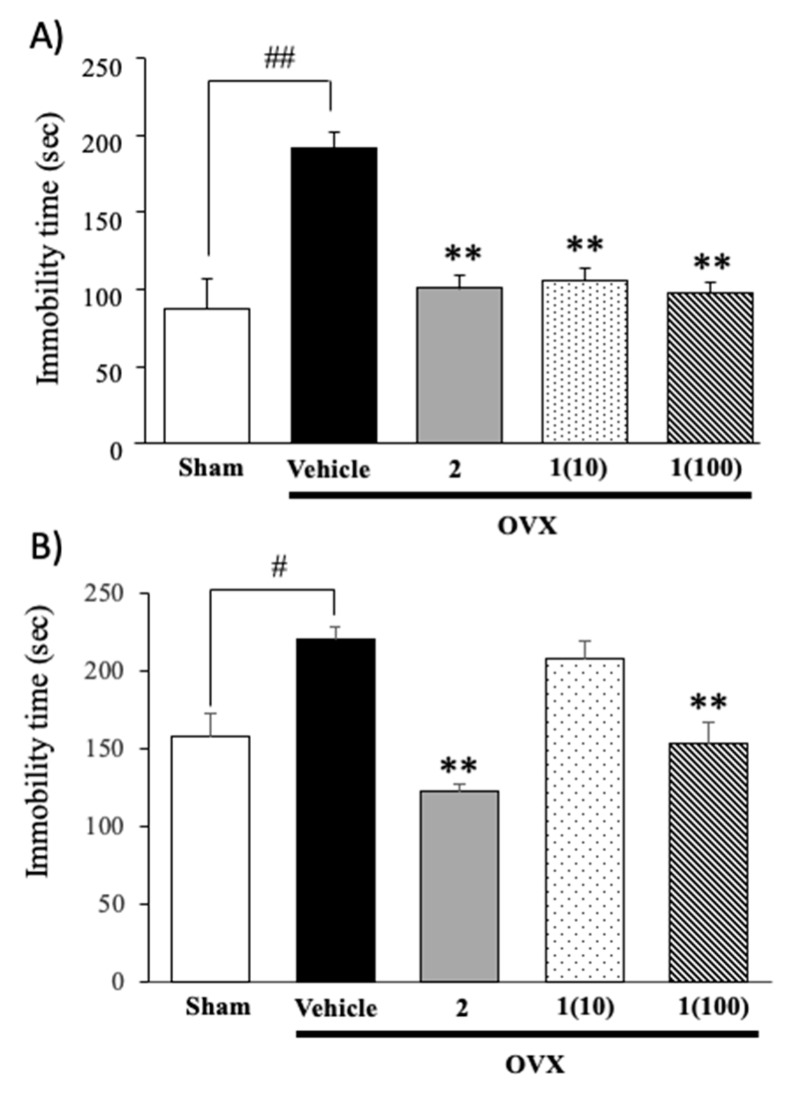
The effect of puerarin (**1**) and 17β-estradiol (**2**) on ovariectomy-induced hopeless behaviors in TST (**A**) and FST (**B**). Each column represents the mean ± SEM (*n* = 8–12). ** *p* < 0.001 vs. the vehicle-treated OVX group. # *p* < 0.05, ## *p* < 0.001 vs. the sham-operated group.

The OVX group treated with vehicle exhibited significantly longer immobility times in the TST and FST than the sham-operated group, indicating that ovariectomy induced depressive-like behavior. Treatment of OVX groups with 17β-estradiol (**2**) and puerarin (**1**) resulted in significantly shorter immobility times than those in the vehicle-treated control OVX mice (Figure 2) (for detailed statistical analysis, see Supplementary Materials, Tables S1 and S2).

**Figure 3:**
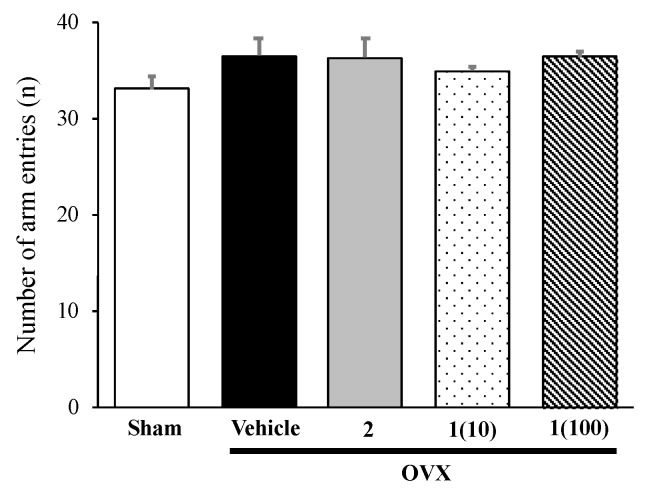
The effect of puerarin (**1**) and 17β-estradiol (**2**) on the locomotor activity in the Y-maze task. The number of arm entries of each group was determined. Each column represents the mean ± SEM (*n* = 8–10 in each animal group).

To exclude false-positive results, a Y-maze task was also performed, to evaluate the locomotor activity. Results showed that neither 17β-estradiol (**2**) nor puerarin (**1**) altered the locomotor activity of OVX mice (Figure 3).

### 2.2. Changes in Uterine Weights and Volumes after the Administration of Puerarin (***1***) to OVX Animals

As summarized in Table 1, ovariectomy caused a significant decrease in uterine weight and volume in OVX mice compared with those with the sham operation. Treatment with puerarin (**1**), at 100 mg/kg, and with 17β-estradiol (**2**), at 1 µg/kg, significantly attenuated the effects of ovariectomy on uterine weight and volume (Table 1) (for detailed statistical analysis, see Supplementary Materials, Tables S3 and S4).

### 2.3. Administration of Puerarin (***1***) Reverses the Ovariectomy-Induced Increase in Serum Corticosterone Levels

**Figure 4:**
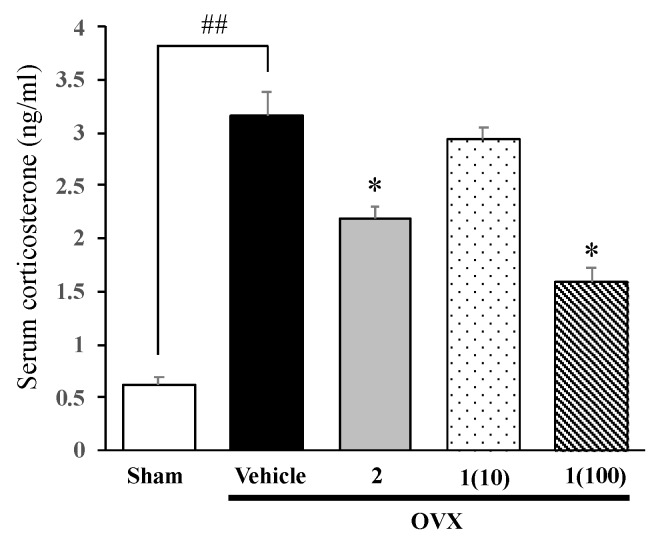
Effects of puerarin (**1**) and 17β-estradiol (**2**) on ovariectomy-induced elevations in serum corticosterone levels. Each column represents the mean ± SEM (n = 3–5). ^##^ *p* < 0.001 vs. the sham-operated group. * *p* < 0.05 vs. the vehicle-treated OVX group.

Serum corticosterone levels were measured in order to clarify whether the feedback mechanism regulating endocrine secretion via the HPA axis was impaired by ovariectomy (Figure 4). Vehicle-treated OVX mice had significantly higher serum corticosterone levels than sham-operated mice. However, ovariectomy-induced elevations in corticosterone levels were significantly suppressed by the daily treatment with 17β-estradiol (**2**), at 1 µg/kg, and puerarin (**1**), at 10–100 mg/kg, for 8 weeks (for detailed statistical analysis, see Supplementary Materials, Table S5).

### 2.4. Effects of Puerarin (***1***) and 17β-Estradiol (***2***) on Ovariectomy-Induced Changes in the Hippocampal Expression of Genes Encoding BDNF, ERβ, and ERα

**Figure 5:**
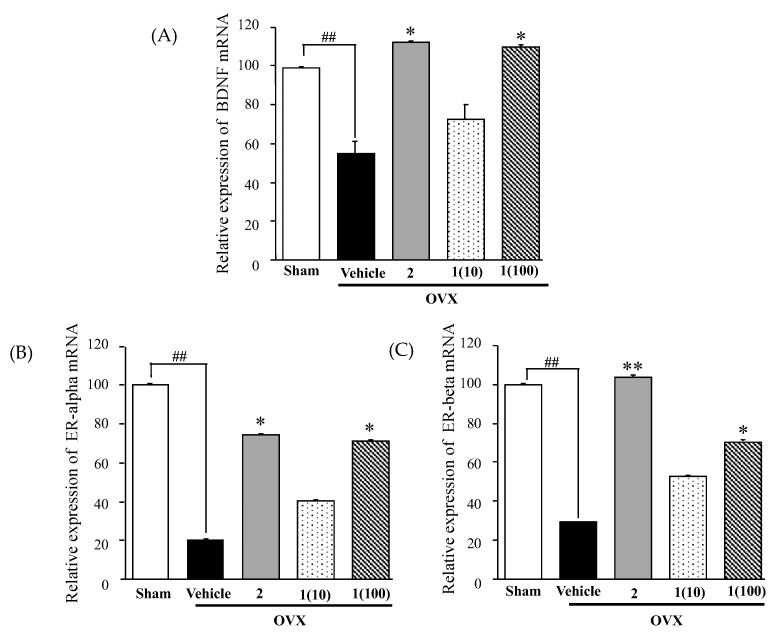
Effects of puerarin (**1**) on ovariectomy-induced alterations in BDNF (**A**), ERα (**B**), and ERβ (**C**) mRNA expression in the hippocampus. The expression level of each mRNA was assessed by QPCR and expressed as a ratio of each mRNA to β-actin mRNA. Each data column represents the mean ± SEM. (*n* = 3–5). ^##^ *p* < 0.001 vs. the sham-operated group. * *p* < 0.05 and ** *p* < 0.001 vs. the vehicle-treated OVX group.

The quantitative real-time PCR (QPCR) study revealed that the expression levels of genes encoding BDNF, ERβ, and ERα in the hippocampus were significantly lower in the OVX group than in the sham-operated group. However, this decrease was reversed by treatment with 17β-estradiol (**2**) (Figure 5). Like 17β-estradiol treatment, the administration of puerarin (**1**) dose-dependently normalized the downregulated expression of these genes in OVX mice (for detailed statistical analysis, see Supplementary Materials, Tables S6–S8).

### 2.5. Puerarin (***1***) Inhibited the Ovariectomy-Induced Suppression of Neurogenesis in the Dentate Gyrus Area in the Hippocampus

**Figure 6:**
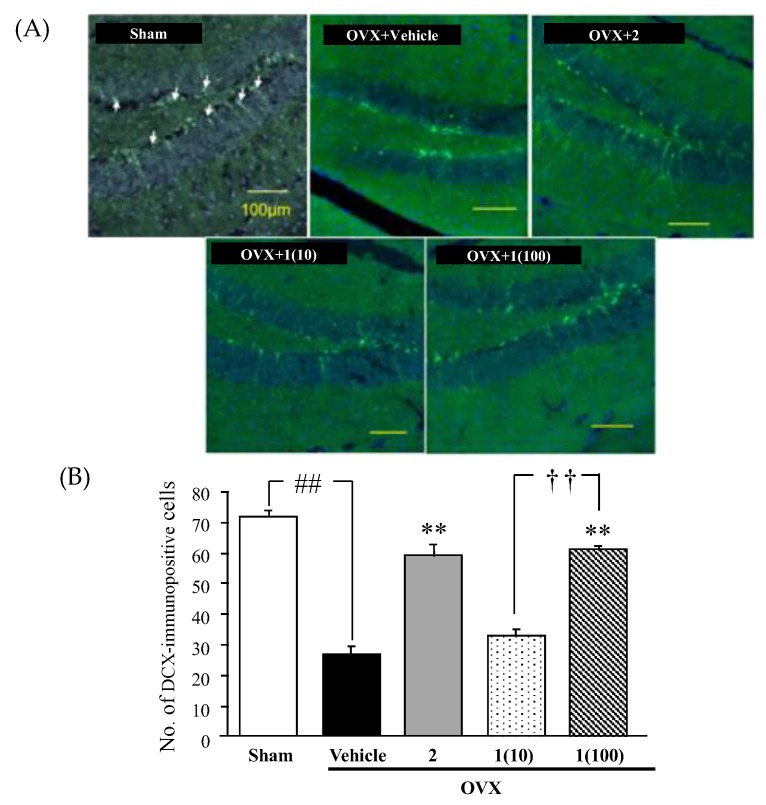
Effects of puerarin (**1**) and 17β-estradiol (**2**) on neurogenesis in the hippocampal dentate gyrus region of the OVX group and the non-OVX group. (**A**) Typical photos showing the immunostaining of doublecortin (DCX)-positive cells in the dentate gyrus. Arrows indicate DCX-immunopositive cells. (**B**) Quantitative analysis of the number of DCX-immunopositive cells among different treatment groups. Each data column represents the mean ± SEM (n = 3–5 in each animal group). ^##^ *p* < 0.001 vs. the sham-operated group. ** *p* < 0.01 vs. the vehicle-treated OVX group. ^††^ *p* < 0.001 between treatments with different doses (for detailed statistical analysis, see Supplementary Materials, Table S9).

The effects of the puerarin (**1**) treatment on the number of doublecortin (DCX)-immunopositive cells in the dentate gyrus of vehicle-treated OVX animals were examined. As shown in Figure 6, the number of DCX-immunopositive cells in the vehicle-treated OVX group was significantly lower than that in the sham-operated group. However, daily treatment with puerarin (**1**), as well as with 17β-estradiol (**2**), significantly reversed ovariectomy-induced decreases in the number of DCX-immunopositive cells.

## 3. Discussion

The present study aimed to investigate the effects of puerarin (**1**), a major constituent of a Thai medicinal plant *P. candollei* var. *mirifica*, on the depressive-like behavior in an estrogen-deficient OVX model of menopause, as well as to elucidate its mechanisms of action. The results obtained demonstrated that, like 17β-estradiol (**2**), puerarin (**1**), at the dose of 100 mg/kg/day, ameliorated ovariectomy-induced depressive-like behavior in mice, suggesting that these effects involve improvements in the hyperactivated function of the HPA axis, as well as downregulated the expression of neurotrophin and neurogenesis in the hippocampus in OVX animals.

We first investigated the effects of puerarin (**1**) on immobility in OVX animals in the TST and FST as an index of hopelessness or despair, which is a major symptom exhibited by patients with depression and menopause. This type of symptom is characterized by an increase in immobility times in the TST and FST, and it is used as an index of depressive-like behavior in rodents. OVX mice exhibited the depressive-like symptoms in both FST and TST when compared with sham-operated mice. Ovariectomy is known as “surgical menopause”, and it is accompanied by a decrease in estrogen. It has been reported that estradiol increases serotonin uptake in the frontal cortex and the hypothalamus in OVX rats [15]. Estrogen also reduced monoamine oxidase (MAO) activity, thus increasing the levels of serotonin in the brain. Moreover, estrogen has also been shown to have a neuroprotective effect, which prevents degeneration of neurons and promotes the neurogenesis [16]. Therefore, these findings confirm the effects of the deletion of ovarian hormones on the induction of depressive behaviors. Early and repeated administration of puerarin (**1**) and 17β-estradiol (**2**) ameliorated OVX-induced depressive-like behavior in the TST and FST. Interestingly, in the present study, it was found that the effective dose (≤10 mg/kg, p.o.) of puerarin (**1**) to reduce the immobility in the TST was apparently lower than its effective dose (≤100 mg/kg, p.o.) in the FST. The reason for the different susceptibility to puerarin (**1**) in the FST and TST is still unknown. However, this effect may be due to a deep hypothermia caused by the immersion in the FST, which is necessary to produce a behavioral despair, while this effect does not occur in the TST. Steru et al. [17] found that the TST displays a different spectrum of pharmacological sensitivity from that of FST procedures, thus providing more sensitivity to a lower dose of drugs. In addition, Chatterjee et al. [18] reported that the underlying pathophysiology of behavioral despair in the FST and TST is different, and that the FST, and not the TST, should be used as a model for negative symptom of psychosis in mice. Taken together, the obtained results allow us to speculate about how the mechanism by which puerarin (**1**) improved the behavioral despair in the TST differs from the mechanism by which this compound exerted the anti-depressive action in the FST.

In order to clarify the mechanism(s) by which the treatment with puerarin (**1**) ameliorated depressive-like symptoms in OVX animals, the hypertrophy of the uterus in OVX animals was examined. A hypertrophy of the uterus is a classical endpoint that is commonly and widely used as an indirect measurement of the estrogenic potency of endocrine-disrupting chemicals in the OVX rodent model [10]. In the present study, both 17β-estradiol (**2**) and puerarin (**1**), at high doses, were found to significantly increase uterine weights and volumes in OVX animals, suggesting that puerarin (**2**) exhibits estrogenic-like activity, in a similar manner to 17β-estradiol (**2**). Our finding is in agreement with the previous reports which demonstrated that puerarin (**2**) exerted uterotrophic effects in OVX mice after long-term treatment [19,20]. Moreover, in the MCF-7 cell proliferation assay, puerarin (**2**) was found to bind to both estrogen receptors (ERs) and exhibited estrogenic activity [21]. The uterotrophic effects of puerarin (**2**) are attributed, in part, to the activated expression of three estrogen-responsive genes in the uterus, i.e., insulin-like growth factor 1 (IGF-1), complement protein 3 (C3), and progesterone receptor (PR). In turn, stimulation of the uterine IGF-1 activates the proliferation of myometrium and endometrium [22]. Furthermore, ovariectomy-induced decreases in ERα and ERβ mRNA expression levels in the hippocampus were reversed by the administration of 17β-estradiol (**2**), as well as a high dose of puerarin (**1**). In OVX animals, the only glandular source of estrogens is the adrenal gland [23], which secretes a small amount of estradiol. Estradiol may also be synthesized in hippocampal tissue [24], where it plays an important role in synaptic plasticity [25] and neuroprotection [26]. The long-term deprivation of estrogen induces a decrease in the expression levels of ER in the hippocampus [27]. Based on these findings, the results obtained in this study indicate that the presence of extra-gonadal sources of estrogens or puerarin (**1**) could contribute to the functional significance of upregulated hippocampal ERα in the absence of gonadal hormones. In addition to the hormonal regulation of ERs, their functions may be modulated by extracellular signals [28], such as IGF-1 and epidermal growth factor (EGF), which are known to activate ERs, thereby increasing the expression of ER target genes [29].

It was found, in the present study, that OVX mice showed significantly higher serum corticosterone levels than in the sham-operated group, suggesting that the HPA axis is activated in OVX animals. Ovariectomy-induced changes in serum corticosterone levels remain controversial since previous studies have demonstrated that ovariectomy exerted no effect on serum corticosterone levels or actually decreased the levels when compared to the control. The reasons for this discrepancy are still unexplained; however, our results showed that ovariectomy-induced elevations in serum corticosterone levels were accompanied by depressive-like behavior, which is similar to stress-induced endocrine responses and behavioral changes. Mizuki et al. [30] reported that exposure to chronic mild stress, an experimental model of depression, markedly elevated serum corticosterone levels and induced depressive-like behavior in mice, and these elevations were reversed by a repetitive treatment with antidepressant drugs. Previous findings indicate also that patients with depression show a hyperactivity of the HPA axis, which is attributable to an impaired feedback mechanism of the HPA axis [31,32].

Administration of puerarin (**1**) and 17β-estradiol (**2**) significantly attenuated the effects of ovariectomy-induced elevations in serum corticosterone levels to the same extent. The exact mechanisms underlying the effects of puerarin (**1**) are still unclear; however, a speculative explanation is that puerarin (**1**) may prevent ovariectomy-induced dysfunctions in a negative feedback mechanism of the HPA axis. This hypothesis is supported by previous findings which demonstrated that 17β-estradiol (**2**) interfered with the activity of neurotransmitters regulating the secretion of corticotropin-releasing factor (CRF) from the hypothalamus. Lund et al. [33] demonstrated that physiological doses of 17β-estradiol (**2**) or diarylpropionitrile (DPN), an ERβ selective agonist, increased neuronal activities in upstream inhibitory regions such as the medial prefrontal cortex (mPFC), thereby increasing inhibitory outflow from the mPFC to the paraventricular nucleus (PVN), from which CRF is secreted from CRF neurons and regulates the activity of the HPA axis. This finding was in agreement with Oyala et al., who demonstrated that the administration of the biologically active *S*-enantiomer of diarylpropionitrile (*S*-DPN) significantly reduced anxiety-like behaviors in OVX wild-type mice, but not in mutant female mice carrying a null mutation for ERβ gene [34]. Moreover, in vitro studies showed that puerarin (**1**) acts as a phytoestrogen that binds to ER, particularly to the ERβ subtype with a relatively higher affinity than to ERα [35]. Thus, puerarin (**1**) seems to suppress ovariectomy-induced elevations of serum corticosterone levels via the same mechanism as estrogen, which involves ERβ, thereby exerting antidepressant-like actions in OVX animals.

Another potential antidepressant mechanism of puerarin (**1**) and estrogen involves the upregulation of BDNF mRNA expression in the hippocampus. The neurotrophin BDNF has been shown to play an important role not only in the hippocampal synaptic plasticity, the neurobiological basis of learning and memory, but also in emotional performance, such as depression, both of which are mediated by synaptogenesis [36], neurogenesis [37,38], changes in morphologies of cells and dendrites [39], and a modulation of the efficacy of neurotransmitter release [40]. Previous studies showed that patients with depression, as well as various animal models of depression, had reduced BDNF levels in the hippocampus. The present results revealed that ovariectomy downregulated the expression of BDNF mRNA in the hippocampus in a manner that was reversed by estradiol replacement therapy, which is consistent with previous findings [41,42]. Sohrabji et al. [41] reported that the gene-encoding BDNF and the canonical estrogen response element found in estrogen-target genes contain similar sequences, and that the estrogen replacement increases the BDNF expression in many brain regions, including the hippocampus in OVX rats through interaction with the estrogen responsive element-like portion on the BDNF gene. Zhou et al. [43] suggested that estradiol modulated the expression of BDNF as a consequence of the activation of the cyclic AMP response element-binding protein (CREB) via protein kinases, such as protein kinase A, calcium/calmodulin-dependent protein kinase type IV (CaMK IV), and mitogen-activated protein kinase (MAPK). In addition, long-term exposure to high concentrations of corticosterone can decrease the BNDF level and impaired hippocampal neurogenesis [44]. Our results also showed that treatment with puerarin (**1**) decreased the serum corticosterone level in OVX mice, leading to the regulation of the BDNF mRNA expression in the hippocampus. Therefore, the upregulatory effects of puerarin (**1**) on the BDNF mRNA expression in the hippocampus may result from a direct interaction between puerarin (**1**) and the estrogen response element-like sequence on the BDNF gene and/or with the activation of signaling between protein kinases and CREB and the effect of puerarin (**1**) on the HPA axis.

We have also found in this study that ovariectomy reduced the number of DCX-immunopositive cells in the dentate gyrus region of the hippocampus. DCX is a neuron-specific protein that promotes microtubule polymerization and the stabilization of microtubules in early mitotic neurons. DCX-immunopositive cell is generally used to determine immature or young neurons as an index of neurogenesis [45]. A previous study demonstrated that hippocampal neurogenesis was impaired in animal models of depression and was susceptible to a stress-related paradigm, as well as to antidepressant drug treatments [30]. Therefore, the decreased number of DCX-immunopositive cells in the hippocampus of OVX animals indicates a reduction of neurogenesis, and may be relevant to the depressive-like behavior observed in these animals. This assumption was supported by the fact that administration of 17β-estradiol (**2**) and puerarin (**1**), at the dose of 100 mg/kg/day, was able to reverse ovariectomy-induced impairments in hippocampal neurogenesis, resulting in an improvement of the depressive-like behavior caused by ovariectomy. The results indicated that puerarin (**1**), in high dose, possessed the estrogenic activity similar to that of 17β-estradiol (**2**), although, with a weaker effect. Long-term treatment of puerarin (**1**) ameliorated depressive-like behaviors of OVX mice by affecting the HPA-axis, estrogen receptor, and neurogenesis in the hippocampus. Interestingly, in the present study, we demonstrated that, in order to achieve a similar effect to 17β-estradiol, it is necessary to increase the dose of puerarin (**1**) by 100,000-fold. Therefore, the underlying mechanisms may be involved not only in the suppression of HPA axis hyperactivity, decreasing corticosterone serum levels and mimicking estrogenic-like activity through the ERα and ERβ subtypes but also in non-estrogen receptor-mediated mechanisms. Therefore, further investigation is needed to clarify this effect.

## 4. Materials and Methods

### 4.1. General Experimental Procedures

Melting point was determined on a Bock monoscope and was uncorrected. Optical rotation was measured on an ADP410 Polarimeter (Bellingham + Stanley Ltd., Tunbridge Wells, Kent, UK). ^1^H and ^13^C NMR spectra were recorded at ambient temperature on a JEOL JNM-a 400 instrument (JEOL Ltd., Tokyo, Japan), operating at 400 and 100 MHz, respectively, and chemical shifts were given in *δ* (ppm), using residual solvent peaks (DMSO-_d6_) as references. FABMS data were obtained by using a JEOL JMS 700 mass spectrometer (JEOL Ltd., Tokyo, Japan) and *m*-nitrobenzyl alcohol matrix (Sigma-Aldrich, St. Louis, MO, USA). A Merck (Darmstadt, Germany) silica gel GF_254_ was used for preparative TLC, and a Merck Si gel 60 (0.2–0.5 mm) was used for column chromatography.

### 4.2. Plant Materials and Isolation of Puerarin (***1***)

The tuberous root bark of *Pueraria candollei* var. *mirifica* (Airy Shaw & Suvat.) Niyomdham (Family Fabaceae) was collected in Ubon Ratchathani Province, Northeastern Thailand, in March 2010. The plant material was identified by Dr. Thaweesak Juengwatanatrakul, Faculty of Pharmaceutical Sciences, Ubon Ratchathani University, Ubon Ratchathani Province, Thailand. A voucher specimen (NI-PSKKU 007–010) was deposited at the Herbarium of the Faculty of Pharmaceutical Sciences, Khon Kaen University, Thailand. The dried powdered bark (10 kg) was extracted with hexane (3 × 45 L) and filtered with Whatman No. 1 filter paper. The dried residue was extracted with EtOAc (3 × 60 L), and the process was repeated with EtOH (3 × 60 L). The EtOH extracts were combined and evaporated under reduced pressure, to give 226 g of a crude ethanol extract, which was subsequently dissolved in H_2_O (450 mL), with the assistance of an ultrasonic bath. The aqueous solution of the extract was applied on a column of Diaion HP-20 (2.26 kg, Mitsubishi Chemical Corp, Tokyo, Japan) and eluted with H_2_O (20 × 2 L), MeOH–H_2_O [(1:1), 15 × 2 L], MeOH–H_2_O [(3:1, 10 × 2 L)], MeOH (8 × 2 L), and EtOAc (5 × 2 L). The fractions eluted MeOH–H_2_O (1:1) were combined and evaporated under reduced pressure, to give a crude aqueous methanol extract (55.3 g), which was applied over a column of silica gel (1.1 kg) and eluted with mixtures of CHCl_3_–MeOH and CHCl_3_–MeOH–H_2_O, wherein 250 mL fractions were collected as follow: Fractions (Frs.) 1–3 (CHCl_3_–MeOH, 9:1), 4–20 (CHCl_3_–MeOH, 17:3), 21–25 (CHCl_3_–MeOH, 4:1), 26–54 (CHCl_3_–MeOH–H_2_O, 4:1:0.1), 55–63 (CHCl_3_–MeOH–H_2_O, 6.5:3.5:0.1), 64–68 (CHCl_3_–MeOH, 1:1), and 69 (MeOH). Frs. 37–42 were combined (15.53 g) and purified by reversed-phase high-performance liquid chromatography (HPLC) of the JASCO model 887-PU pump and a 875 UV variable-wavelength detector (JASCO International Co. Ltd., Tokyo, Japan), using TSKgel ODS-80TS column (5 um, 6 i.d. × 60 cm × 2, Tosoh Chemicals Co. Ltd., Tokyo, Japan) with an isocratic elution using a mixture of MeCN–H_2_O (3:17, *v*/*v*) as a mobile phase, at a flow rate of 45.0 mL/min, to obtain 2.98 g of puerarin (**1**) (retention time, *t*_R_ = 130 min, Supplementary Material, Figure S1), which was further recrystallized in ethanol to yield 2.65 g of puerarin (**1**). Puerarin (**1**) was identified by comparison of its MS, ^1^H, and ^13^C NMR data (Supplementary Materials, Figures S2–S4) to those reported in the literature, [46] as well as by co-elution on the TLC with the authentic sample.

### 4.3. Animals

Sixty 5-week-old female ICR mice (Japan SLC Inc., Shizuoka, Japan) were used in the experiments. Animals were housed in paper-bedding cages and given food and water ad libitum. Housing conditions were a 12 h light–dark cycle (lights on: 07:00 –19:00 h) under temperature control (24 ± 1 °C) and constant humidity (65 ± 2%). All behavioral experiments were performed between 07:30 and 17.00 h, and each animal was used once, according to the Guiding Principles for the Care and Use of Animals (NIH Publications # 8–23, revised in 2011) and were also approved by the Institutional Animal Use and Care Committee of the University of Toyama, Japan.

### 4.4. Surgical Procedure

**Figure 7:**
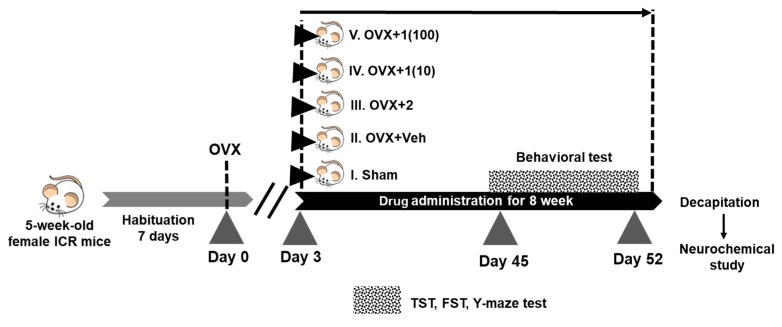
A schematic representation of the experimental protocols. The mice were divided into two main groups, i.e., the sham-operated group and the OVX group. The OVX group was divided into four groups, which daily received (i) vehicle, (ii) 17*β*-estradiol (**2**), (iii) **1** (10 mg/kg), and (iv) **1** (100 mg/kg) for 8 weeks. Behavioral tests started on day 45 until day 52. One day after behavioral tests, the mice were decapitated, and the blood, uterus, and brain were collected for neurochemical determination.

Ovariectomy was conducted under pentobarbital anesthesia (Nembutal^®^: 60 mg/kg; Ceva Sante Animale, Libourne, France), as previously described [47]. Briefly, the exposed ovary and associated oviduct were removed, and skin incisions were closed. The sham-operated group underwent the same procedure without removal of the ovaries. After a 3-day recovery period, the OVX mice were randomly divided into four groups; OVX + vehicle, OVX + 17β-estradiol (1 µg/kg/day) [OVX + **2**], OVX + puerarin 10 mg/kg/day [OVX + **1** (10)], and OVX + puerarin 100 mg/kg/day [OVX + **1** (100)]. There were 8–12 animals in each group. The body weights of the mice were recorded weekly, during the experimental period. The experimental protocols are summarized in Figure 7.

### 4.5. Drug Administration

The vehicle (corn oil, J-oil Mills, Inc., Tokyo, Japan) and drugs were administered daily (Figure 7), at 8.00 a.m., for 8 weeks. On a behavioral-testing day, all treatments were conducted 1 h before testing. The mice were divided into five groups. The 1st group was a sham-operated group (corn oil, po). The 2nd group was an OVX group (corn oil, po). The 3rd group was the OVX, which received 1 µg/kg (ip) of 17*β*-estradiol (**2**) (Sigma-Aldrich, St. Louis, MO, USA). The 4th and 5th groups were OVX groups, which received, respectively, 10 and 100 mg/kg (po) of puerarin (**1**). A small volume of absolute ethanol (25 uL) was added to 0.5 g of puerarin (**1**), and then corn oil (25 mL) was added in order to give a final concentration of 20 mg/mL, and it was used as a stock solution. The reference standard drug, 17*β*-estradiol (**2**), was suspended in corn oil. The stock solutions were kept in a dark bottle, at room temperature, until used.

### 4.6. Behavioral Studies

Mice were placed in the testing room for 1 h prior to the behavioral test for habituation. The order of the behavioral tests was as follow: Y-maze test (day 45), FST (pre-swimming on day 47–48, test session on day 48–49), and TST (day 51–52). One day after completion of the behavioral tests, the mice were decapitated. All tissues were collected immediately and kept at −80 °C throughout the experiment.

#### 4.6.1. Locomotor Test

In order to exclude false positive results from drug-induced hyperlocomotion in hopeless behavioral tests, locomotor activity was performed, using the Y-maze task. The Y-maze consisted of three equal arms that were 40 cm long, 18 cm high, and 3 cm wide at the bottom, and 12 cm wide at the top. The arms were positioned at equal angles, in a Y-shape. One hour after drug administration, the mice were individually placed on one arm, and the total arm entries were recorded manually, over an 8 min period, for measuring the locomotion activity [48].

#### 4.6.2. Forced Swimming Test (FST)

The FST is the method used to examine “learned helplessness and hopeless” behaviors in rodent models. Mice were placed individually in a transparent glass cylinder (12 cm in diameter and 25 cm high), which was filled with water, to the height of 10 cm, at 25 ± 2 °C. Twenty-four hours before the test, a pre-swimming test session was performed for 15 min. In the test session, each mouse received a vehicle or a drug treatment 1 h before the test. Immobility time was recorded during the 5 min period. Mice were considered to be immobile when they discontinued struggling and remained floating motionless in the water, making only those movements necessary to keep their head above the water.

#### 4.6.3. Tail Suspension Test (TST)

The TST was developed as a rodent screening test for potential (human) antidepressant drugs. This test was conducted as previously described [49]. Briefly, 1 h after the administration of drugs, each mouse was individually suspended 50 cm above the floor by adhesive tape placed approximately 1 cm from the tip of the tail. Animal behavior in the test was video-recorded for later analyses. Mice were considered immobile when they hunk passively and completely motionless with a movement speed of no more than 0.05 cm^2^/s, monitored by the SMART^®^ system version 2.5 (PanLab, S.L., Barcelona, Spain). Immobility times were recorded for the last 4 min during a 6 min period.

### 4.7. Uterine Hypertrophy Test

A uterine hypertrophy test was performed, to examine whether the 8-week treatment of OVX mice with puerarin (**1**) exerts in vivo estrogenic effects. The weight and volume of the uterus, isolated from each group, was directly measured on a scale and recorded.

### 4.8. Neurochemical Studies

#### 4.8.1. Measurement of Serum Corticosterone Levels

After completing behavioral studies, mice received the last dose of drugs prior to decapitation. Animals were anesthetized, using 60 mg/kg pentobarbital, after which they were transcardially perfused with 0.1 M phosphate buffer saline (PBS, pH = 7.4), and the blood samples were collected. Samples were left overnight at room temperature and then centrifuged at 3000 rpm, at 4 °C, for 15 min. The supernatant was collected and frozen at –80 °C, until used. Assay Max Corticosterone ELISA kit (Assaypro LLC, St. Charles, MO, USA) was employed to measure the serum corticosterone levels according to the previously described procedure [49]. The serum sample (25 µL) was added in 96-well microplates of ELISA kit. Then, 25 µL of biotinylated protein was immediately added to each well, gently mixed, and incubated for 2 h, at room temperature, and then washed with 200 µL of washing solution for five times, and streptavidin–peroxidase conjugate (50 µL) was added into each well before incubation at room temperature for 30 min. Each well was washed with washing solution (200 µL) five times and then added to chromogen substrate (50 µL) and incubated for 20 min, at room temperature. All of the reactions were stopped by adding 50 µL of stop solution. The absorbance was immediately determined at 450 nm.

#### 4.8.2. Quantitative Real-Time Polymerase Chain Reaction (QPCR)

Mouse BDNF, CREB, ERα, and ERβ expression levels were analyzed by QPCR. Total RNA from the hippocampus was extracted, using TRIzol^®^ reagent (Invitrogen, Rockville, MD, USA), according to the manufacturer’s instructions. First-strand cDNA was synthesized with oligo (dT) primers and M-MLV reverse transcriptase (Invitrogen, Rockville, MD, USA) and used as a template for QPCR. QPCR was conducted by using SsoAdvanced™ Universal SYBR^®^ Green Supermix (BioRad, Hercules, CA, USA). The following primers were synthesized by Macrogen (Seoul, South Korea): amplification was performed by using the following gene-specific PCR primer sets as follows: (1)-β-actin: 5′-AAC GGT CTC ACG TCA GTG TA-3′ (sense) and 5′-GTG ACA GCA TTG CTT CTG TG-3′ (antisense); (2)-BDNF: 5′-GAC AAG GCA ACT TGG CCT AC-3′ (sense) and 5′-CCT GTC ACA CAC GCT CAG CTC-3′ (antisense); (3)-CREB: 5′-TAC CCA GGG AGG AGC AAT AC-3′(sense) and 5′-GAG GCA GCT TGA ACA ACA AC-3′ (antisense); (4)-ER-β: 5′-CAG TAA CAA GGG CAT GGA AC-3′ (sense) and 5′-GTA CAT GTC CCA CTT CTG AC-3′ (antisense); and (5)-ER-α: 5′-GAC CAG ATG GTC AGT GCC TT-3′ (sense) and 5′-ACT CGA GAA GGT GGA CCT GA-3′ (antisense). A melting curve analysis of each gene was performed each time after amplification. β-Actin mRNA was used as a control to which results were normalized. Relative expression was used to represent data.

### 4.9. Analysis of Neurogenesis in the Dentate Gyrus

Doublecortin (DCX)-immunopositive cells in the hippocampal dentate gyrus region were measured as a marker of neurogenesis and immature neurons in the present study. After completing behavioral studies, animals were intracardiacally perfused with 4% paraformaldehyde (PFA) in PBS, under pentobarbital anesthesia (whole brains were removed and fixed in 4% PFA in PBS for 24 h). PBS with 30% sucrose was used as sucrose fixation for cryo-protection at 4 °C for 24 h, and then the brains were fixed on dry ice and stored at –80 °C, until sectioned. Coronal brain sections (10 µm) were sliced on a cryostat (Leica CM3050, Nussloch, Germany), fixed on glass slides, and stored at –20 °C, until stained. Brain slides were washed with PBS, and then antigen activation was achieved by incubating in Target Retrieval Solution (Dako Japan Inc., Kyoto, Japan), at 98 °C, for 30 min, and 10% normal goat serum in PBS was used as a blocking solution at room temperature for 1 h. Slides were then incubated with an anti-DCX antibody (rabbit polyclonal antibody 1:1000 dilution, ab18723; Abcam, Cambridge, UK) at 4 °C for 3–6 h. Slides were washed with PBS six times, and then incubated with a goat anti-rabbit IgG antibody (1:300; Alexa Fluor 594, A11037, Invitrogen, Tokyo, Japan) for 1 h. Brain slides were mounted with 4′,6-diamidino-2-phenylindole (DAPI)-containing medium. Images of brain slices were captured by using an upright optical microscope, under a 20 x objective (BX-61, Olympus, Tokyo, Japan) equipped with a digital camera (DP50, Olympus, Tokyo, Japan). DCX-immunopositive cells were counted in the dentate gyrus area at –2.06 mm, relative to the bregma (2–3 sections for each animal), in a blind manner.

### 4.10. Statistical Analysis

Data were expressed as the mean ± SEM and analyzed by a one-way analysis of variance (ANOVA), followed by Tukey’s test for multiple comparisons among different groups. Differences with *p* < 0.05 were considered significant. SigmaStat^®^ version 3.5 (SYSTAT Software Inc., Richmond, CA, USA) was used for statistical analyses.

## 5. Conclusions

The present study provides corroborative evidence, for the first time, that puerarin (**1**), a glycosyl isoflavone isolated from the root bark of the Thai medicinal plant *Pueraria candollei* var. *mirifica*, relieves a depressive-like behavior in OVX mice, by suppressing HPA axis hyperactivity, decreasing corticosterone serum levels, and mimicking estrogenic-like activity through the ERα and ERβ subtypes. Furthermore, puerarin (**1**) promotes hippocampal neurogenesis, possibly by upregulating BDNF mRNA expression, and increases the number of DCX-immunopositive cells. Thus, puerarin (**1**) has the potential for treatment of depression in estrogen-deficient women.

## Figures and Tables

**Table 1:** Effects of puerarin (**1**) and 17β-estradiol (**2**) on uterine weight and volume.

Treatment	Uterine Weight/Body Weight (g/kg)	Uterine Volume/Body Volume (cm^3^/kg)
Sham-operated	7.68 ± 0.31	10.32 ± 1.39
OVX + vehicle	1.66 ± 0.11 ^##^	1.37 ± 0.31 ^##^
OVX + 2	5.50 ± 0.50 **	4.79 ± 0.23 *
OVX + 1 (10 mg/kg)	1.57 ± 0.15 ^‡^	2.35 ± 0.34
OVX + 1 (100 mg/kg)	3.15 ± 0.48 **	4.94 ± 0.81 ^*^

Data were expressed as mean ± SEM (*n* = 8–9). ^##^ *p* < 0.001 vs. the sham-operated group. * *p <* 0.05, ** *p* < 0.001 vs. the vehicle-treated OVX group. ^‡^ *p* < 0.05 between treatments with different doses.

## Supplementary Materials

The following are available online, Table S1. One-way analysis of variance (ANOVA) test of TST. Table S2. One-way analysis of variance (ANOVA) test of FST. Table S3. Statistical analysis of the effect of puerarin (**1**) and 17β-estradiol (**2**) on uterine weight. Table S4. Statistical analysis of the effect of puerarin (**1**) and 17β-estradiol (**2**) on uterine volume. Table S5. Statistical analysis of the effect of administration of puerarin (**1**) on serum corticosterone levels. Table S6. Statistical analysis of the effects of puerarin (**1**) and 17β-estradiol (**2**) on the hippocampal BDNF mRNA expression. Table S7. Statistical analysis of the effects of puerarin (**1**) and 17β-estradiol (**2**) on the hippocampal ERα mRNA expression. Table S8. Statistical analysis of the effects of puerarin (**1**) and 17β-estradiol (**2**) on the hippocampal ERβ mRNA expression. Table S9. Statistical analysis of the effects of puerarin (**1**) on the neurogenesis in the dentate gyrus area in the hippocampus. Figure S1. HPLC chromatogram of puerarin (**1**) from root bark of *Pueraria candollei* var. *mirifica*. Figure S2. ^1^H NMR spectrum of puerarin (**1**) (400 MHz, DMSO-*d_6_*). Figure S3. Expansion of the ^1^H NMR spectrum of puerarin (**1**) (400 MHz, DMSO-*d_6_*). Figure S4. ^13^C NMR spectrum of puerarin (**1**) (100 MHz, DMSO-*d_6_*).
