# Supplementary material for: Effects of Puerarin on the Ovariectomy-Induced Depressive-Like Behavior in ICR Mice and Its Possible Mechanism of Action
Source: Molecules. 2019 Dec 13;24(24):4569. doi: 10.3390/molecules24244569 (PMC6943479; doi:10.3390/molecules24244569)

Supplementary Materials

Effects of Puerarin on the Ovariectomy-Induced Depressive-Like Behavior in ICR Mice and Its Possible Mechanism of Action

Ariyawan Tantipongpiradet[1], Orawan Monthakantirat[1], Onchuma Vipatpakpaiboon[1], Charinya Khampukdee[2], Kaoru Umehara[3], Hiroshi Noguchi[3], Hironori Fujiwara[4], Kinzo Matsumoto[4], Nazim Sekeroglu[5], Anake Kijjoa[6*], Yaowared Chulikhit[1*]

[1] Division of Pharmaceutical Chemistry, Faculty of Pharmaceutical Sciences, Khon Kaen University, Khon Kaen 40002, Thailand; ariyawanps@gmail.com (A.T.); oramon@kku.ac.th (O.M.); onchuma.aim@gmail.com (O.V.); yaosum@kku.ac.th (Y.Ch.).

[2] Division of Research and Development, Faculty of Pharmaceutical Sciences, Khon Kaen University, Khon Kaen 40002, Thailand; charkh@kku.ac.th (C.K.)

[3] Department of Pharmacognosy, School of Pharmaceutical Sciences, University of Shizuoka, Yada 52-1, Shizuoka-shi, Shizuoka 422-8526, Japan; umehara@u-shizuoka-ken.ac.jp (K.U.); noguchi@u-shizuoka-ken.ac.jp (H.N.).

[4] Division of Medicinal Pharmacology, Institute of Natural Medicine, University of Toyama, 2630 Sugitani, Toyama 930-0194, Japan; arawijuf@inm.u-toyama.ac.jp (H.F.); mkinzo@inm.u-toyama.ac.jp (K.M.)

[5] Department of Horticulture, Faculty of Agriculture, Killis 7 Aralik University, Killis 79000, Turkey; nsekeroglu@gmail.com (N.S.)

[6] *ICBAS*-Instituto de Ciências Biomédicas Abel Salazar and CIIMAR, Rua de Jorge Viterbo Ferreira, 228, 4050-313 Porto, Portugal; ankijjoa@icbas.up.pt (A.K.)

*Correspondence: yaosum@kku.ac.th (Y.Ch.) and ankijjoa@icbas.up.pt (A.K.), Tel.: +351-220428331 (A.K.)

Table S1. One-way analysis of variance (ANOVA) test of TST.

Group comparison	One-way analysis of varience	
	P	F ($DF_{between\ group}$, $DF_{residual}$)
Sham *vs.* OVX	<0.001	*F*(4,36)=13.095
OVX *vs.* **2**	<0.001	
OVX *vs.* **1**(10)	<0.001	
OVX *vs.* **1**(100)	<0.001	

Table S2. One-way analysis of variance (ANOVA) test of FST.

Group comparison	One-way analysis of varience	
	P	F ($DF_{between\ group}$, $DF_{residual}$)
Sham *vs.* OVX	0.002	*F*(4,36)=14.729
OVX *vs.* **2**	<0.001	
OVX *vs.* **1(**100)	<0.001	
OVX + **1**(10) *vs.* OVX +**1**(100)	0.008	

Table S3. Statistical analysis of the effect of puerarin (**1**) and 17β-estradiol (**2**) on uterine weight.

Group comparison	One-way analysis of varience	
	P	F ($DF_{between\ group}$, $DF_{residual}$)
Sham *vs.* OVX	<0.001	*F*(4,36)=60.016
OVX *vs.* **2**	<0.001	
OVX vs. **1**(100)	0.025	
OVX + **1**(10) *vs.* OVX + **1**(100)	0.020	

Table S4. Statistical analysis of the effect of puerarin (**1**) and 17β-estradiol (**2**) on uterine volume.

Group comparison	One-way analysis of varience	
	P	F ($DF_{between\ group}$, $DF_{residual}$)
Sham *vs.* OVX	<0.001	*F*(4,22)=19.134
OVX *vs.* **2**	0.048	
OVX *vs.* **1**(100)	0.047	

Table S5. Statistical analysis of the effect of administration of puerarin (**1**) on serum corticosterone levels.

Group comparison	One-way analysis of varience	
	P	F ($DF_{between\ group}$, $DF_{residual}$)
Sham *vs.* OVX	<0.001	*F*(4,13)=27.707
OVX vs. 2	0.047	
OVX *vs.* **1**(100)	0.021	

Table S6. Statistical analysis of the effects of puerarin (**1**) and 17β-estradiol (**2**) on the hippocampal BDNF mRNA expression.

Group comparison	One-way analysis of varience	
	P	F ($DF_{between\ group}$, $DF_{residual}$)
Sham +OVX	<0.001	-
OVX + **2**	0.006	$F(4,15)=5.795$
OVX + **1** (100)	0.012	

Table S7. Statistical analysis of the effects of puerarin (**1**) and 17β-estradiol (**2**) on the hippocampal ERα mRNA expression.

Group comparison	One-way analysis of varience	
	P	F ($DF_{between\ group}$, $DF_{residual}$)
Sham + OVX	<0.001	
OVX + **2**	0.002	$F(4,14)=24.135$
OVX + **1** (100)	0.008	

Table S8. Statistical analysis of the effects of puerarin (**1**) and 17β-estradiol (**2**) on the hippocampal ERβ mRNA expression.

Group comparison	One-way analysis of varience	
	P	F ($DF_{between\ group}$, $DF_{residual}$)
Sham +OVX	<0.001	-
OVX + **2**	<0.001	$F(4,14)=24.135$
OVX + **1** (100)	0.003	

Table S9. Statistical analysis of the effects of puerarin (**1**) on the neurogenesis in the dentate gyrus area in the hippocampus.

Group comparison	One-way analysis of varience	
	P	F ($DF_{between\ group}$, $DF_{residual}$)
Sham *vs.* OVX	<0.001	
OVX *vs.* **2**	<0.001	$F(3,11)=46.83$
OVX *vs.* **1** (100)	<0.001	
OVX+**1**(10) *vs.* OVX+**1**(100)	<0.001	

Figure S1. HPLC chromatogram of puerarin (**1**) from root bark of *Pueraria candollei* var. *mirifica.*

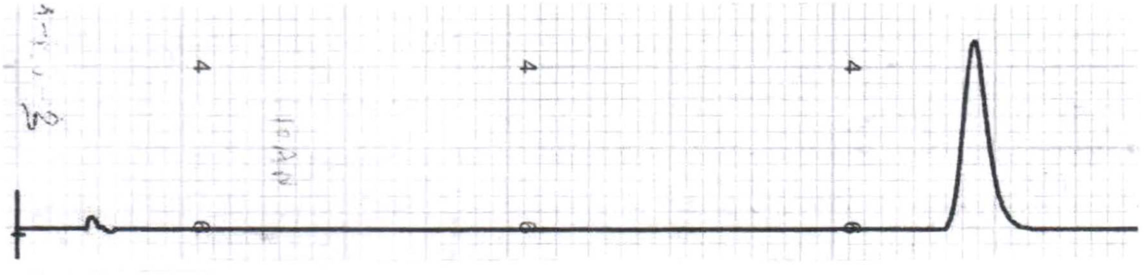

Figure S2. ^{1}H NMR spectrum of puerarin (**1**) (400 MHz, DMSO-*d6*)

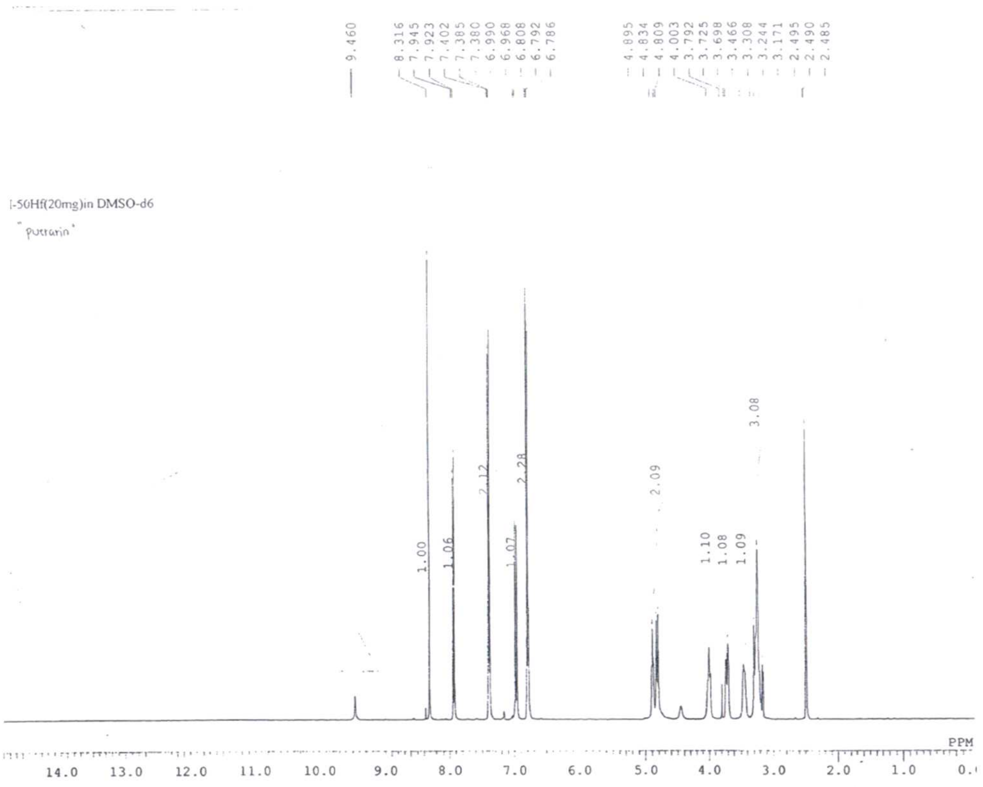

Figure S3. Expansion of the ^{1}H NMR spectrum of puerarin (**1**) (400 MHz, DMSO-*d6*)

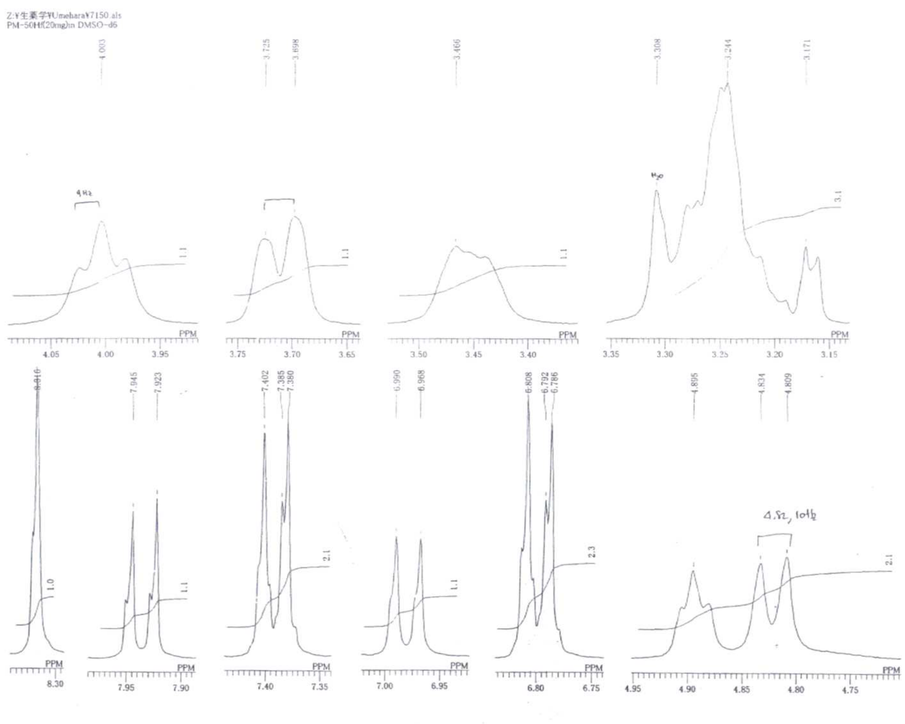

Figure S4. ^{13}C NMR spectrum of puerarin (**1**) (100 MHz, DMSO-*d6*)

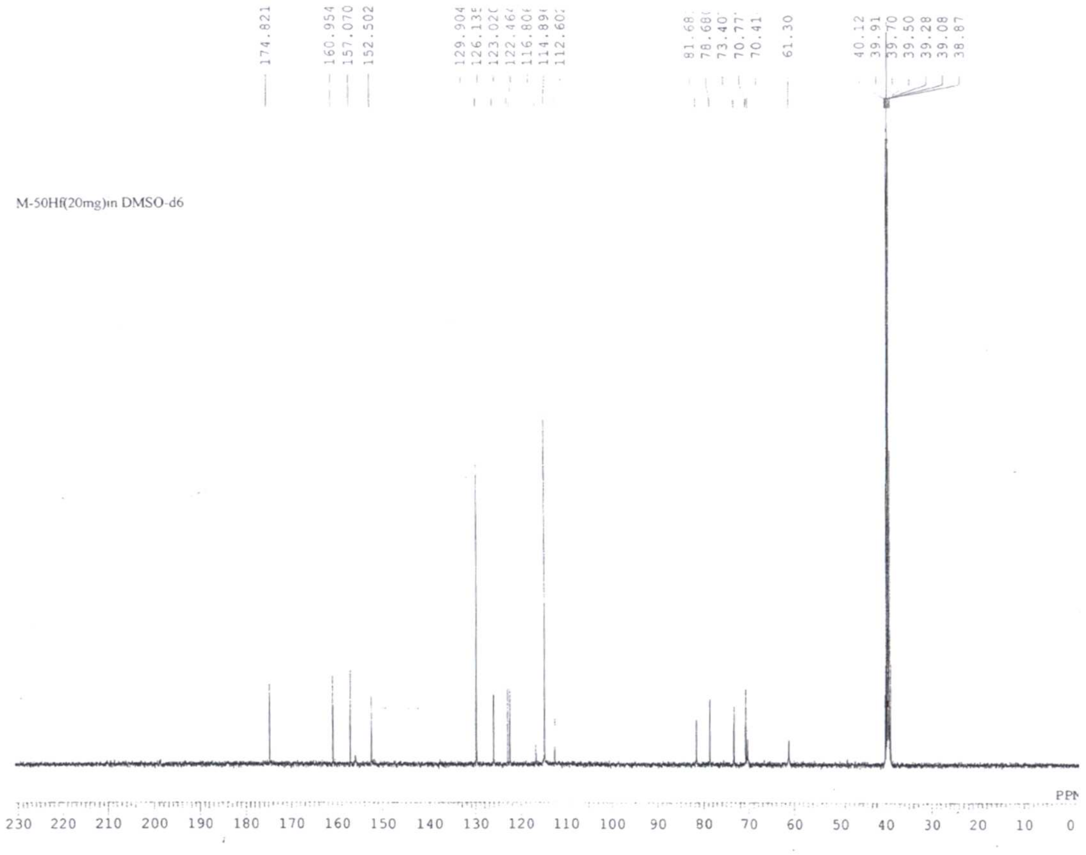

Supplement: Supplementary file 1 [file molecules-24-04569-s001.pdf]
